# Transfer Learning for Sentiment Analysis Using BERT Based Supervised Fine-Tuning

**DOI:** 10.3390/s22114157

**Published:** 2022-05-30

**Authors:** Nusrat Jahan Prottasha, Abdullah As Sami, Md Kowsher, Saydul Akbar Murad, Anupam Kumar Bairagi, Mehedi Masud, Mohammed Baz

**Affiliations:** 1 Department of Computer Science and Engineering, Daffodil International University, Dhaka 1341, Bangladesh; jahannusratprotta@gmail.com; 2 Department of Computer Science & Engineering, Chittagong University of Engineering & Technology, Chattogram 4349, Bangladesh; abdullahassami@gmail.com; 3 Department of Computer Science, Stevens Institute of Technology, Hoboken, NJ 07030, USA; 4 Faculty of Computing, Universiti Malaysia Pahang, Pekan 26600, Malaysia; saydulakbarmurad@gmail.com; 5 Computer Science and Engineering Discipline, Khulna University, Khulna 9208, Bangladesh; anupam@ku.ac.bd; 6 Department of Computer Science, College of Computers and Information Technology, Taif University, P.O. Box 11099, Taif 21944, Saudi Arabia; mmasud@tu.edu.sa; 7 Department of Computer Engineering, College of Computers and Information Technology, Taif University, P.O. Box 11099, Taif 21944, Saudi Arabia; mo.baz@tu.edu.sa

**Keywords:** sentiment analysis, Bangla-BERT, transfer learning, transformer, word embedding, Bangla NLP

## Abstract

The growth of the Internet has expanded the amount of data expressed by users across multiple platforms. The availability of these different worldviews and individuals’ emotions empowers sentiment analysis. However, sentiment analysis becomes even more challenging due to a scarcity of standardized labeled data in the Bangla NLP domain. The majority of the existing Bangla research has relied on models of deep learning that significantly focus on context-independent word embeddings, such as Word2Vec, GloVe, and fastText, in which each word has a fixed representation irrespective of its context. Meanwhile, context-based pre-trained language models such as BERT have recently revolutionized the state of natural language processing. In this work, we utilized BERT’s transfer learning ability to a deep integrated model CNN-BiLSTM for enhanced performance of decision-making in sentiment analysis. In addition, we also introduced the ability of transfer learning to classical machine learning algorithms for the performance comparison of CNN-BiLSTM. Additionally, we explore various word embedding techniques, such as Word2Vec, GloVe, and fastText, and compare their performance to the BERT transfer learning strategy. As a result, we have shown a state-of-the-art binary classification performance for Bangla sentiment analysis that significantly outperforms all embedding and algorithms.

## 1. Introduction

Sentiment classification is the process of examining a piece of text to forecast how an individual’s attitude toward an occurrence or perspective will be oriented. The sentiment is usually analyzed based on text polarity. Typically, a sentiment classifier categorizes positive, negative, or neutral [1]. Sentiment extraction is the backbone of sentiment categorization, and considerable study has been conducted. The next crucial step is sentiment mining, which has increased tremendously in recent years in line with the growth of textual data worldwide. People now share their ideas electronically on various topics, including online product reviews, book or film studies, and political commentary. As a result, evaluating diverse viewpoints becomes essential for interpreting people’s intentions. In general, sentiment refers to two distinct sorts of thought, either positive or negative, across several platforms where mass opinion has worth.

For example, internet merchants and food suppliers constantly enhance their service in response to customer feedback. For instance, Uber or Pathao, Bangladesh’s most popular ride-sharing service, leverages consumer feedback to improve its services. However, the difficulty here is traversing through the feedback manually, which takes far too much time and effort. Automatic Sentiment Detection (ASD) can resolve this issue by categorizing the sentiment polarity associated with an individual’s perspective. This enables more informed decision-making in the context of one’s input. Additionally, it may be utilized in various natural language processing applications, such as chatbots [2].

As a result of numerous revolutionary inventions and the persistent efforts of researchers, the area of NLP has arisen. Deep Learning (DL) approaches have been increasingly popular in recent years as processing power has increased and the quantity of freely accessible data on the Web has increased. As word embedding improves the efficiency of neural networks and the performance of deep learning models, it has been used as a foundation layer in a variety of deep learning methods.

Earlier attempts to implement sentiment analysis in Bangla have relied on non-contextualized word embeddings (Word2Vec and fastText), which present a series of static word embeddings without considering many other contexts in which they could occur. However, the Bidirectional Encoder Representations from Transformers’s (BERT) recent advent phenomenon tremendously amplifies the contextualization strategy [3]. As the trend switched toward transformer-based architectures consisting of attention heads, BERT has established itself as the most impressive NLP model capable of performing superbly in any NLP operation with proper fine-tuning for specific downstream tasks. BERT is a pre-trained state-of-the-art (SOTA) language model that is highly bidirectional and has been trained on a large English Wikipedia corpus [4]. For 104 languages, there is a generic mBERT model [5]. Since it does not do well on other language tasks, the researchers developed their language-specific BERT model that performs pretty similarly to the original BERT model. Consequently, we employ the superior BERT model for Bangla sentiment analysis.

Bangla is spoken by around 250 million people and is the world’s fifth most widely spoken language. However, due to a scarcity of resources, pre-trained models such as transformer-based BERT were unsuitable for any task. This issue was handled by developing a monolingual Bangla BERT model for the Bangla language. To obtain the best possible result for this sentiment analysis dataset, we fine-tuned the Bangla-BERT (https://huggingface.co/Kowsher/bangla-bert (accessed on 1 February 2022)) model which had been trained on the largest BanglaLM dataset (https://www.kaggle.com/datasets/gakowsher/bangla-language-model-dataset (accessed on 1 February 2022)) [6] and then set connection to a Convolutional Neural Network (CNN) and Long Short Term Memory (LSTM).

This research examined an extensive sentiment dataset, including reviews from various domains like the internet and social networking sites, including politics, sports, products, and entertainment. To do this at first, we fine-tuned the BERT, and then the aggregating layer has been utilized as the text embedding; finally, we have developed a deeply integrated model such as CNN-BiLSTM for decision-making. Here, we showed two kinds of comparison of our proposed work: the first one is word embedding techniques such as Word2Vec, GloVe, fastText with BERT, and the second one compares various machine learning and deep learning algorithms to ensure the best performance of hybrid integrated model CNN-BiLSTM. to ensure the best performance of hybrid integrated model CNN-BiLSTM. This work will assist merchants in rapidly integrating a classification model into their own systems for the purpose of tracking customer feedback. The following points can notate the main contribution of this paper:

- This work has ensured the hybrid integrated model such as CNN-BiLSTM, and it has been used in combination with monolingual BERT to address the issue of sentiment analysis in Bangla;
- We compared Word2vec, GloVe, FastText, and BERT, where we demonstrated how transformer architecture exceeds all prior state-of-the-art approaches and becomes the new state-of-the-art model with proper fine-tuning;
- To do this, we developed a Bangla pre-trained BERT model for transfer learning (Huggingface: Kowsher/bangla-bert).

The following Section 2 discusses related work. Section 3 presents our proposed methodology, while Section 4 and Section 5 discuss the embedding and classification algorithms. We reported the results in Section 6 and concluded with a discussion and recommendations for further study in Section 7.

## 2. Related Work

Sentiment Analysis is a well-known problem that involves assessing the polarity of an individual’s viewpoint. The SA procedure entails extracting features from a text corpus, building a classifier model, and assessing its performance [7]. This type of usual procedure has been applied to a variety of sentiment classification tasks, including categorization of movie reviews [8], online product reviews [9], and Twitter tweets [10]. Akshay et al. [11] developed a model for detecting positive and negative sentiments in restaurant reviews, with a maximum accuracy of 94.5%. Another analysis revealed an accuracy of 81.77% for smartphone reviews when the researchers employed SVM as a classifier [12]. Sentiment analysis on Twitter data for the Portuguese language has been defined in [13]. Ombabi et al. demonstrated a sentiment classifier for the Arabic language with an accuracy of 90.75%, outperforming the state of the art [14]. The blending of two languages to create a new one is a regular occurrence in NLP. Such work has been conducted on vernacular Singaporean English, a product of the coalescence of Chinese and Malay languages [15]. However, the majority of efforts on sentiment categorization focus on English and other widely spoken languages. The biggest constraint on Bengali sentiment analysis research is a lack of appropriate resources and datasets. Numerous deep learning techniques have been developed for a variety of domains, including microblogs, product reviews, and movie reviews. To classify sentiment polarity on those domains, SVMs use maximum entropy [16] and Multinomial Naive Bayes (MNB) [17] have been utilized. Hossain et al. [18] created a Bangla book review dataset, applied machine learning approach, and discovered that MNB had an accuracy of 88%. Similar study using SVM on the Bangladesh cricket dataset achieved 64.60% accuracy [19]. Sarker et al. suggested a sentiment classifier for Bengali tweets that outperforms n-gram and SentiWordnet features by 45%. The sentiment categorization of Bengali film reviews demonstrates a range of performance values when using various machine learning techniques. Amongst them, the SVM and LSTM models achieve 88.89 and 82.41% accuracy, accordingly [20]. Pre-trained language models have notably become pivotal in a variety of NLP applications since they can leverage massive amounts of unlabeled data to obtain general language representations; Elmo [21], GPT [22], and BERT [4] are just a few of the possible best example. Among them, the BERT model receives the most attention due to its unmatched bidirectionality and attention mechanism. As a result, researchers are tracking its effect on downstream NLP tasks. Since BERT is trained exclusively in English, researchers create their language-specific BERT model to get higher precision on the task since it has been demonstrated that language-specific BERT models outperform generic mBERT models. Recent research has also shown outstanding task performance in sentiment analysis [23,24] attempting to uncover factors and their related views. Numerous researchers from various countries have developed their respective language BERT models to evaluate the sentiment analysis task. The Arabic BERT model AraBERT scored 99.44 on their distinct sentiment analysis experiment [25], while the Persian ( PersBERT) [26], DutchBERT (BERTje) [27], and Romanian (RobBERT) [28] models scored 88.12, 93.00, and 80.44 on their corresponding sentiment analysis experiments. Russia (RuBERT) [29], China [30], and several other countries develop their language-specific BERT models to obtain greater accuracy across all NLP domains, including sentiment analysis. They compare their model accuracy to that of the mBERT model and discover that their results are significantly higher than their mBERT values. This demonstrates that, while performing sentiment analysis, the monolingual BERT produces the state-of-the-art (SOTA) outcome, discarding all previous attempts and methods.

## 3. Methodology

Though BERT can be used as a feature extraction model, we chose the fine-tuning technique. We have expanded the Bangla-BERT model using two distinct end-to-end deep network layers in this technique: CNN and LSTM.

**Figure 1:**
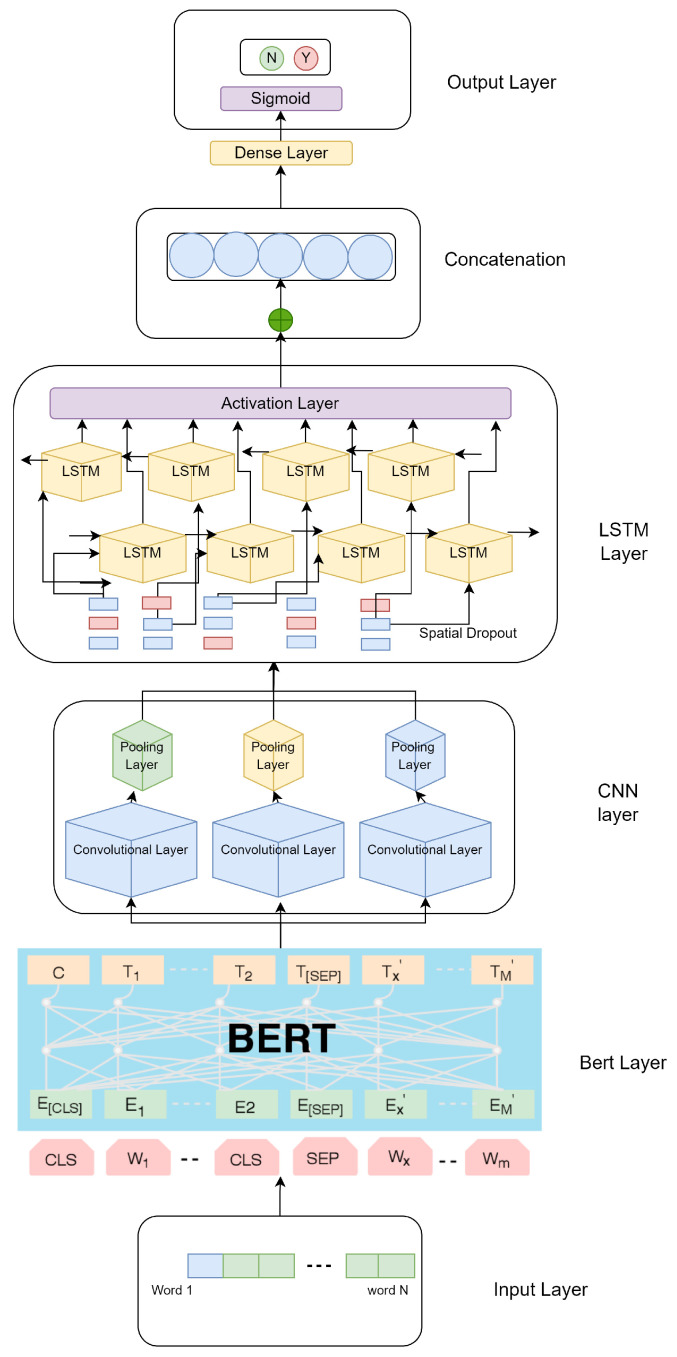
Representative mechanism of Bangla-BERT to CNN-BiLSTM in sentiment analysis. First, BERT accepts tokens for embedding, and then passes through the CNN layer for the extract information. Next, LSTM aids to create a sequence from the extracted information after FNN makes a decision by calculating loss.

BERT generates contextualized embedding vectors for each word that are then fed through one of two deep network layers CNN to LSTM, which has been described in Figure 1. The feature vector is constructed by concatenating the output neurons for each word from the intermediary layer. Then, each vector is processed through a densely linked neural network to reduce its dimension. Softmax is used to classify the final reduced vector. Additionally, three additional learning algorithms with pre-trained word embeddings were incorporated: Word2Vec, Glove, and fastText. Word2Vec has proved to be very effective in the analysis of sentiment in a variety of languages, especially Bengali [31]. Meanwhile, fastText has gained widespread interest in Bengali text analysis, owing to its action on n-grams at the word level [32].

**Figure 2:**
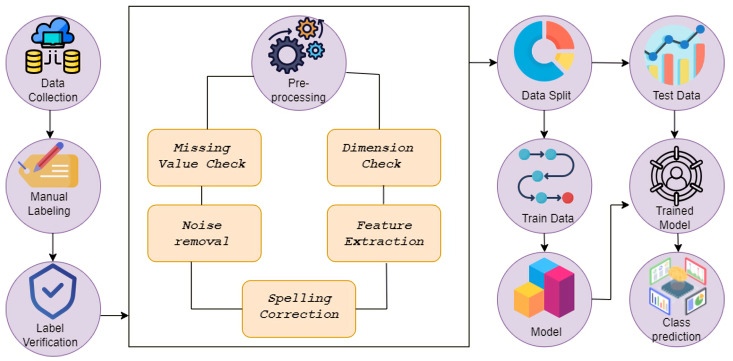
Whole workflow of sentiment analysis. The first phase is data collecting and then labelling, Secondly, the data have been pre-processed and the last phase is decision-making by modeling.

Data gathering and labeling were the initial steps in this classification work. The data acquired from social media was carefully labeled. A relevant domain expert validated the manual labeling. The data were then subjected to a pre-processing approach that included the removal of missing values, noise, and spelling correction, as well as feature extraction and dimension reduction. Figure 2 illustrates the entire process of this research. Following that, the data set was partitioned into training and test segments at a 7:3 ratio. We trained and evaluated the model using supervised learning. The trained model was then fed the testing data, and the prediction accuracy was compared to the ground truth. The whole methodology of this work has been depicted in Figure 2.

### 3.1. Data Source

We gathered data for the corpus from a range of sources, including internet sites and social networking sites where individuals share their valued opinions. A substantial portion of the data was gathered via Facebook, Twitter, and YouTube comments. Apart from that, online stores have grown to be a significant part of digital marketing. As a result, we also gathered data from online retailer product reviews. Additionally, certain film and book reviews have been included in this corpus. Table 1 presents an overview of the dataset.

### 3.2. Data Collection

**Figure 3:**
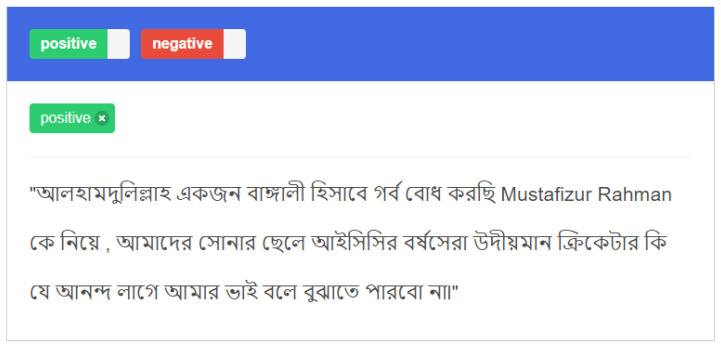
Snapshot of the text annotation tool.

Following that, we have collected a total of 8952 samples from referred sources, where 4325 samples are positive and the rest of the samples are negative. For this sample labeling, ten native speakers annotated it using the web annotation software doccano. Each participant annotated 30% of the dataset individually by assigning positive or negative labels. We then applied kappa statistics to labeling data collectors and the majority voting of the labeling by the native speaker group. The annotation tool is depicted in Figure 3.

### 3.3. Data Preprocessing

Data preparation is essential in machine learning-based classification, as the model’s accuracy is heavily dependent on the quality of the input data [33]. We employ this procedure to prepare data for machine utilization. The next subsection describes the many procedures involved in data preprocessing.

#### 3.3.1. Missing Value Check

We began our data processing phase by addressing the dataset’s missing values. We’ve encountered two distinct sorts of missing values. Several of these are data omissions, while others provide less information than is required. If all information was absent, we eliminated the entire sample by erasing the entire row. If there was insufficient information, the value was manually adjusted using a similar observation value.

#### 3.3.2. Noise Removal

After correcting for missing values, we enhanced the dataset by removing noise from the samples. Non-Bangla letters or characters, meaningless special symbols, and emoticons are all considered noise. Though emoticons can express a wide variety of emotions, we have seen that only a small percentage of data contains emoticons. As a result, the cleaning operation includes the removal of emoticons. Table 2 illustrates the processing steps with an example.

#### 3.3.3. Spelling Correction

Since the data were gathered from various people, some words may have been mistyped or misspelled. We used the Bangla Academy’s [34] available dictionary (AD) database to determine the most suitable structure of the word. From the sentiment corpus, SC=d1,d2,d3,…dn where d1 is the text data. Each *v* that does not appear in AD is deemed a misspelled word. The right word was then obtained from AD and substituted for the incorrect one. Table 2 details the workflow used to analyze the sample data.

#### 3.3.4. Feature Extraction

Feature extraction, alternatively referred to as word embedding, represents words in such a way that related terms are translated appropriately [35]. We employed four distinct word extraction approaches in this analysis to examine which word extraction technique performs the best on Bangla language sentiment.

We explored the most commonly used methods for word embedding, including Word2Vec, GloVe, fastText, as well as the state-of-the-art model BERT. We trained Word2Vec, fastText, and GloVe to demonstrate more incredible performance using the skip-gram model rather than the CBOW model as it can better represent fewer recurring words. In Section 4, we widely described the feature extraction techniques in detail.

## 4. Encoding Algorithm

We used the preprocessed data for the word embedding algorithm’s training model [36]. We examined the performance of each model independently using a variety of window sizes, vector sizes, and iterations over the dataset. The models we developed were created with the Gensim tool, which is an efficient toolkit for performing a variety of typical natural language processing tasks and includes a Word2Vec, fasttext, Glove models’ implementation, whereas, to train BERT, we have used the Huggingface open-source tool.

### 4.1. Word2Vec

Word2Vec is an extensively used word embedding method. It uses a neural network to ascertain the semantic similarity of the context of the words [37]. Word2Vec implements two inversely related architectures: a continuous bag of words (CBOW) and a Skip-Gram. Skip-Gram is an architecture for unsupervised learning used to discover semantic concepts depending on their context [38]. Skip-Gram works based on Equation (1) to get the maximum average logarithmic probability:

(1) $$E=-\frac{1}{v}\sum_{v=1}^{V}\sum_{-c\leq m\leq c,m\neq 0}\log\log\left[p\left(w_{v+m}|w_{v}\right)\right].$$

It makes use of the provided training $w_{1},w_{2},w_{3}\ldots w_{N}.$*c* denotes the context size, also known as the window size. *E* is the embedding size. The probability (wn+m|wn) can be calculated using Equation (2):

(2) $$p\left(o\right)=\frac{exp(u_{i}^{T}.u_{o}^{{}^{\prime }})}{\sum_{v\in V}expexp\left(u_{z}^{T}.u_{o}^{{}^{\prime }}\right)}$$

Here, *V* represents the vocab list and *u* signifies ’input’ and $u^{\prime }$ is the ’output’ vector representations of *i*, *o* accordingly. CBOW forecasts the target word using the semantic information available in a collection of given text [39]. It makes use of distributed continuous contextual representations. CBOW constructs a static window from a word sequence. Then, using a log-linear classifier trained on upcoming and prior words, the model assumes the window’s middle word. The greater the value of Equation (3), the more likely the word wt will be inferred:

(3) $$\frac{1}{V}\sum_{v\in V}\log\left(p\left(w_{v-c},\ldots w_{v-2},w_{v-1}{,}_{v+1}{,}_{v+2}\ldots w_{v+c}\right)\right).$$

**Figure 4:**
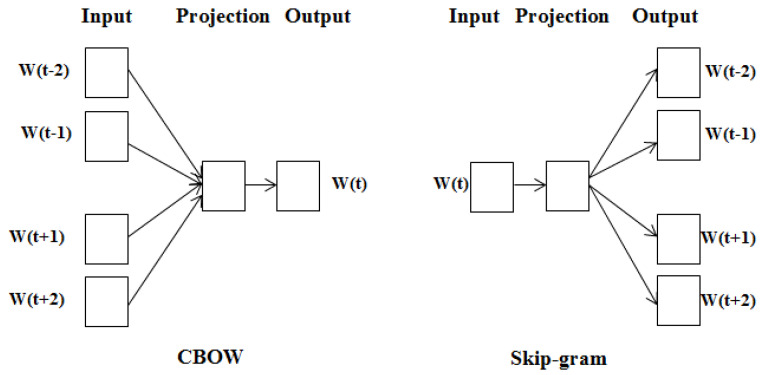
CBOW and Skip−Gram architecture.

Here, *V* and *c* are equivalent to the Skip-Gram model parameters. Figure 4 illustrates both models.

### 4.2. GloVe

GloVe or Global Vectors imply word embeddings relying on their co-occurrence [40]. The co-occurrence matrix indicates the frequency with which a specific pair of words occurs. The matrix of co-occurrences, designated as *C*, in which the rows and columns correspond to the vocabulary of words. Each element in *C*, i.e., $C_{ij}$, indicates the frequency with which the word occurs. Increased weight results in an increase in vector similarity.

### 4.3. FastText

FastText is a highly robust algorithm for word embedding that takes advantage of subword information [41]. This model learns the embeddings from the training words’ character n-grams. As a result, during the training period, a non-existent word in the vocabulary can be created from its constituent n-grams. This resolves the constraint of Word2Vec and GloVe, which require training to obtain a non-vocab word.

The first matrix of weights, *A*, is a look-up table for the words. After averaging the word representations, a text representation is created, which is subsequently input into a linear classifier. The text representation is a protected variable that could be utilized. This structure is identical to Mikolov’s cbow model [42], except that the intermediate word is substituted by a tag. They estimate the likelihood function over the predefined set of classes using the softmax activation function *f*. This results in a reduction of the negative log-likelihood over the classes for a set of *N* documents:

(4) $$-\frac{1}{N}\sum_{n=1}^{N}y_{n}\log\left(f\left(BAx_{n}\right)\right),$$

where $x_{n}$ is the nth document’s normalized bag of information, $y_{n}$ is its label, and *A* and *B* are its weight matrices. Concurrently, on many CPUs, this model is trained to employ a stochastic gradient descent and a linearly decreasing learning rate.

### 4.4. BERT

BERT is the world’s first pre-trained bidirectional and entirely unsupervised language representation approach, having been trained on a massive English Wikipedia corpus [4]. It is an Open-Source Language Representation Model developed by Google AI. Prior to training, BERT can read the texts (or a series of words) in either direction, which is superior to a single-direction technique. BERT surpasses all other word embedding algorithms with fine-tuning, attaining state-of-the-results (SOTA) in multiple NLP applications. BERT employs Transformer, an attention method that discovers semantic aspects of speech (or sub-words) in a text.

The attention mechanism of the transformer is the core component of BERT. The attention mechanism helps extract the semantic meaning of a term in a sentence that is frequently tied to its surroundings. The context information of a word serves to strengthen its semantic representation [43]. Simultaneously, other terms in the context frequently play multiple roles in expanding semantic representation. An attention mechanism can enhance the semantic representation of the target sentence by evaluating contextual information.

In contrast to prior word embedding approaches, BERT employs two distinct strategies: masked language modeling (MLM) and next sentence prediction (NSP).

For the purpose of predicting random masked tokens, the Masked Language Model (MLM) is utilized. In addition, 15% of *N* tokens are picked at random for this reason. These are derived by substituting an exclusive [MASK] token for 80% of selected tokens, 10% with a randomized token, and 10% staying unmodified.

In the case of the Next Sentence Prediction (NSP) task, the model is fed pairs of sentences and trained to predict whether the second sentence in the pair corresponds to the succeeding sentence in the original text. According to the original BERT research, excluding NSP from pre-training can result in a decrease in the model’s performance on specific tasks.

Some research explores the possibilities of leveraging BERT intermediate layers but the most typical is to utilize the last output layer of BERT to boost the efficiency of fine-tuning of BERT.

We compute this sentiment analysis research using a pretrained Bangla-BERT model. This BERT model is comparable to Devlin’s [4] suggested BERT model in terms of performance because it was trained on the largest Bangla dataset yet created. This model demonstrates that state-of-the-art results outperform all preceding results.

The key component of this transformer architecture is the BERT encoder. It is based on a feed-forward neural network and an attention mechanism. Multiple encoder blocks are layered on top of one another to form the Encoder. Each encoder block consists of two feed-forward layers and a self-attention layer that operates in both directions [44].

Three phases of processing are performed on the input: tokenization, numericalization, and embedding. Each token is mapped to a unique number in the corpus vocabulary during the tokenization process, which is known as numericalization. Padding is essential to ensure that the lengths of the input sequences in a batch are similar. When data travel through encoder blocks, a matrix of dimensions (Inputlength)×(Embeddingdimension) for a specific input sequence is provided, providing positional information via positional encoding. The Encoder’s total *N* blocks are primarily connected to obtain the output. A specific block is in charge of building relationships between input representations and encoding them in the output.

The structure of the Encoder is based on multi-head attention. It performs multiple calculations of attention *h* utilizing varying weight matrices and then combines the outcomes [43]. Each of these simultaneous calculations of attention results in the creation of a head. The subscript *i* is used to denote a certain head and its associated weight matrices. Once all of the heads have been calculated, concatenation will proceed. This results in the formation of a matrix with the dimensions $Input\_Length*x(h*d_{v})$. Finally, a linear layer composed of the weight matrix $W^{0}$ with dimensions $(h*d_{v})*Embedding\_dimension$ is added, resulting in an ultimate output with dimensions Input_Length∗Embedding_dimension. In mathematical terms:

$$Multihead\left(Q,K,V\right)=Concat\left(head_{1}^{},\ldots head_{h}\right)W^{0},$$

where $head_{i}=Attention\left(QW_{i}^{Q},KW_{i}^{K},VW_{i}^{V}\right)$ and *Q*, *K*, and *V* are placeholders for various input matrices. Each head is defined by three unique projections (matrix multiplications) determined by matrices in the mechanism of scaled Dot-Product.

$W_{i}^{k}$ with the dimensions $d_{emb\_dim}\times d_{k}$,

$W_{i}^{Q}$ with the dimensions $d_{emb\_dim}\times d_{k}$,

$W_{i}^{v}$ with the dimensions $d_{emb\_dim}\times d_{v}$

The input matrix *X* is projected individually through the above weight matrices to estimate the head. Then, the resultant matrix are as follows::

$XW_{i}^{K}=K_{i}$ with the dimensions input_length $\times d_{k}$

$XW_{i}^{Q}=Q_{i}$ with the dimensions input_length $\times d_{k}$

$XW_{i}^{V}=V_{i}$ with the dimensions input_length $\times d_{v}$

We use these $K_{i}$, $Q_{i}$, and $V_{i}$ to calculate the scaled dot product attention:

(5) $$Attention\left(Q,K,V\right)=softmax\left(\frac{QK^{T}}{\sqrt{d_{k}}}\right)V$$

To assess the similarities of token projections, the dot product of these $K_{i}$ and $Q_{i}$ projections is utilized. Considering $m_{i}$ and $n_{j}$ as the $i^{th}$ and $j^{th}$ token’s projections via $K_{i}$ and $Q_{i}$, Thus, the dot product is as Equation (6):

(6) $$m_{i}n_{j}=cos\left(m_{i},n_{j}\right)\left|\right|m_{i}\left||2|\right|n_{j}\left|\right|2$$

It reflects the relationship between $n_{i}$ and $m_{j}$. Next, for scaling purposes, the resulting matrix is partitioned into elements by the square root of $d_{k}$. The following step entails the row-by-row implementation of softmax. As a result, the matrix’s row value converges to a value between 0 and 1, which equals 1. Finally, $V_{i}$ multiplies this value to obtain the head [4].

## 5. Classification Algorithms

### 5.1. Convolutional Neural Networks (CNN)

CNNs (Convolutional Neural Networks) is a type of deep feed-forward artificial neural network extensively employed in computer vision problems like image classification [45]. CNN was founded by LeCun in the early 1990s [46]. A CNN is a similar multilayer perceptron to a multilayer perceptron (MLP). Because of its unique structure, the model’s architecture allows CNN to demonstrate translational and rotational invariance [47]. A CNN is made up of one or more convolutional layers, associated weights and pooling layers, and a fully connected layer in general. The local correlation of the information is used by the convolutional layer to extract features.

#### 5.1.1. Convolution Layer

The convolution layer uses a kernel to compute a dot product (or convolution) of each segment of the input data, then adds a bias and forwards it through an activation function to build a feature map over the next layer [48,49]. Suppose an input vector for beat samples is $\chi_{i}^{0}=$$\left[x_{1},x_{2},\ldots ,x_{n}\right]$, where *n* is the number of samples/beat. The output values are then calculated using Equation (7):

(7) $$C_{i}^{l,j}=h\left(b_{j}+\sum_{m=1}^{M}w_{m}^{j}x_{i+m-1}^{0j}\right)$$

In this case, *l* is the layer index, *h* is the activation function used to append nonlinearity to this layer, and *b* is the bias term for the *j* feature map. M specifies the kernel/filter size, while w specifies the weight for the jth feature map and *m* filter index.

#### 5.1.2. Batch Normalization

The training data are collected batch by batch. As a result, the batch distributions remain nonuniform and unstable, and therefore must be fitted using network parameters in each training cycle, severely delaying model convergence. To solve this issue, a convolutional layer is followed by batch normalization, an adaptive reparameterization approach. The batch normalization approach calculates the mean $\mu_{D}$ and variance $\sigma_{D}^{2}$ of each batch of training data before adjusting and scaling the original data to zero-mean and unity-variance. Additionally, weight and bias are given to the shifted data ${\hat{x}}_{l}$ to improve its expressive capacity. The calculations are provided by the Equations (8)–(11).

The reparameterization of the batch normalization approach substantially simplifies coordinating updates across layers in the neural network:

(8) $$\mu_{D}=\frac{1}{m}\sum_{i=1}^{m}x_{i}$$

(9) $$\sigma_{D}^{2}=\frac{1}{m}\sum_{i=1}^{m}{\left(x_{i}-\mu_{D}\right)}^{2}$$

(10) $${\hat{x}}_{l}=\frac{x_{i}-\mu_{D}}{\sqrt{\mu_{D}^{2}+\epsilon }}$$

(11) $$y_{i}=\gamma {\hat{x}}_{l}+\beta$$

### 5.2. Max Pooling Layer

The sub-sampling layer is another name for the pooling layer. The proposed method employs the 1D max-pooling layer following the 1D convolutional layer and batch normalization layer, which performs a downsampling operation on the features to reduce their size [48]. It collects small rectangular data chunks and produces a distinct output for each piece. This can be performed in a variety of different methods. In this study, the Maxpooling approach is used to find the largest value in a set of neighboring inputs. The pooling of a feature map inside a layer is defined by (12) [49]:

(12) $$p_{i}^{l,j}=\max_{r\in R}{}_{i\times T+r}^{l,j}$$

The pooling window size is denoted by *R*, while the pooling stride is denoted by *T*. Following that, utilizing several convolutional and max-pooling layers, the obtained features are converted to a single one-dimensional vector for classification. Apart from each classification label corresponding to a single output type, these classification layers are fully coupled. CNN needs fewer experimental parameter values and fewer preprocessing and pre-training methods than other approaches, such as depth and feedforward neural networks [50]. As a result, it is a very appealing framework for deep learning.

### 5.3. Bidirectional Long Short-Term Memory Model

Since deep learning is the most advanced sort of machine learning accessible today, there is an increasing range of neural network models available for use in real-world settings. A successful deep learning method was used in this study to illustrate its unique and exciting problem-solving capabilities. Because of its memory-oriented characteristics, it is known as long short-term memory.

**Figure 5:**
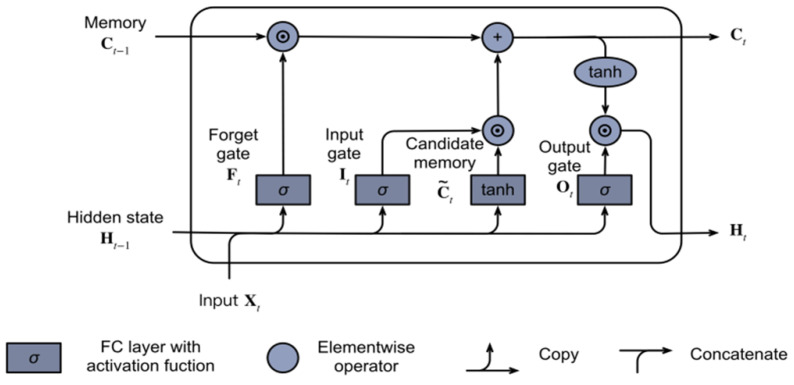
The memory cell of the LSTM layer.

The Bi-LSTM is a deep learning algorithm that analyzes data fast and extracts the critical characteristics required for prediction. This method is an extension of the Recurrent Neural Network methodology (RNN). To tackle the “vanishing gradient” problem of the old RNN structure, the predecessors devised the new network structure of LSTM [51]. The LSTM structure (Cell) has an input gate, an output gate, a forgetting gate, and a memory unit [52]. Figure 5 shows the architecture of the gate.

The math notation of these gates are things such as forget gate ($f_{t}$), input gate ($i_{t}$), and output gate ($O_{t}$) at time *t*. For given input *x* and hidden state $h_{t}$ at time *t*, the computation of lstm alluded to is as below:

$f_{t}=\sigma \left(W_{f}\left[x_{t},h_{t-1}\right]+b_{f}\right)$

$i_{t}=\sigma \left(W_{i}\left[x_{t},h_{t-1}\right]+b_{i}\right)$

$\bar{C_{t}}=\sigma \left(W_{c}\left[x_{t},h_{t-1}\right]+b_{c}\right)$

$C_{t}=i_{t}*\bar{C_{t}}+f_{t}*C_{t-1}$

$O_{t}=\sigma \left(W_{o}\left[x_{t},h_{t-1}\right]+b_{o}\right)$

$h_{t}=O_{t}*tanh\left(c_{t}\right)$

The forget gate in the memory block structure is controlled by a one-layer neural network. The activation of this gate is determined by (13):

(13) $$f_{t}=\sigma \left(W\left[x_{t},h_{t-1},C_{t-1}\right]+b_{f}\right),$$

where $x_{t}$ represents the input sequence, $h_{t-1}$ represents the previous block output, $C_{t-1}$ represents the previous LSTM block memory, and $b_{f}$ represents the bias vector. While σ indicates the logistic sigmoid function, *W* signifies separate weight vectors for each input.

The input gate is a component that uses a basic NN with the tanh activation function and the prior memory block effect to generate fresh memory. These operations are computed using (14) and (15) [53]:

(14) $$i_{t}=\sigma \left(W\left[x_{t},h_{t-1},C_{t-1}\right]+b_{i}\right)$$

(15) $$C_{t}=f_{t}\cdot C_{t-1}+i_{t}\cdot \tanh\left(\left[x_{t},h_{t-1},C_{t-1}\right]\right)+b_{c}$$

Long-term dependencies may be avoided by deliberately constructing and remembering long-term information, which is the default behavior of LSTM in practice. The one-way LSTM is based on previous data, which is not always sufficient. Data are analyzed in two directions using the Bi-LSTM. The bi-hidden LSTM’s layer has two values [54], one of which is utilized in forward computation and the other in reverse computation. These two values define BiLSTM’s final output, which tends to improve prediction performance [55].

### 5.4. One-Dimensional CNN-BiLSTM Proposed Method

The one-dimensional CNN (1D CNN) is the same as the classic 2D CNN, other than that the convolution operation is only conducted on one dimension, resulting in a deep architecture as shown in Figure 1. Hence, it can be easily trained on a normal CPU or even embedded development boards [56]. The convolution technique facilitates the development of significant hierarchical features for classification from a dataset. To estimate the dimensions of the output features after 1D CNN, apply the following equation:

(16) $$x=\frac{w+2p-f}{s}+1,$$

where *x* represents the output dimension and *w* represents the size of the input features *f* is the size of the filter used for convolutions, and *p* denotes padding, which is the addition of values to the border before conducting convolution. The variable *s* stands for stride, which is the distance traveled once the convolution procedure is completed.

Because one-dimensional convolution is a linear operation, it cannot be utilized to categorize nonlinear data. The majority of real-world datasets are nonlinear, requiring nonlinear processes after convolution. A nonlinear function is an activation function. The sigmoid, hyperbolic tangent, rectified linear unit (ReLU), and Exponential Linear Unit are the most commonly used activation functions (ELU).

The suggested CNN architecture makes use of the ELU activation function, which is easy to implement and allows for faster processing.

Furthermore, it addresses some of the issues with ReLUs while retaining some of the favorable aspects. It also has no difficulties with gradients vanishing or popping. Finally, the whole method is integrated with BERT. BERT transforms the embedding layer to CNN-BiLSTM for decision-making, which is described in Figure 1.

## 6. Experiment

According to some studies, fine-tuning mBERT with a text classification model generates a lower result than the proposed similar architecture since it eliminates the aggregated weight and restricted data arise and result in improved performance [57]. Another research study reveals that, when a classification algorithm is fine-tuned with BERT, the results are improved to the original BERT fine-tuning approach [58].

The proposed model is a hybrid of BERT and CNN-LSTM. We have used the BERT output as the LSTM’s input. The LSTM layer eventually extracts features from BERT that it has obtained. Then, we have connected CNN to the following layer. As we have used BERT as a sentence encoder, this state-of-the-art model can acquire precise semantic information. Then, we integrated CNN models for text classification. The architecture of Bangla-BERT for sentiment analysis is depicted in Figure 1.

To examine various characteristics of the suggested method, we combined four embedding and thirteen classification methods. In the next section, we have included a table that summarizes the categorization models’ performance. The model with the highest scores has been made publicly available.

### 6.1. Prediction and Performance Analysis

We develop the complete project entirely in Python 3.7. We used Google Collaboratory for GPU support because the data set was substantially larger than average and required the implementation of various deep learning architectures. Sci-kit-learn and Keras (with TensorFlow backend) were utilized for machine learning and DNN frameworks, respectively. Additionally, we included another machine learning framework, Impact Learning.

Having completed the training set, we tuned critical hyperparameters to offer a fine-tuned model with equivalent assessment metrics. Based on the test set, we evaluated the models. We assessed each estimate for accuracy, precision, recall, f1 score, Cohen’s kappa, and ROC AUC. We summarized the findings in Table 3, Table 4, Table 5 and Table 6.

We represented the outcome of Word2Vec embedding with each classification technique in Table 3. As shown in Table 3, the CNN-BiLSTM algorithm achieves the maximum accuracy of 84.93%, while the SVM method achieves the second-highest accuracy of 83.83%. ANN performed the worst, achieving an accuracy of 76.84%. Because CNN-BiLSTM is leading in the F1 score, it is optimum if we implement Word2Vec embedding.

In Table 4, we have used fastText for embedding and used all the algorithms that were used earlier to classify emotions. As seen in the table, CNN-BiLSTM has the highest accuracy of 88.35%, while LDA has the second-highest accuracy of 86.38%. The Naive Bayes classifier performed the worst, with an accuracy of 78.16%. With an F1 score of 85.97%, Impact Learning is the best match when fastText embedding is used.

In Table 5, we used GloVe for embedding and all previous methods for classifying emotions. As can be seen from the table, the CNN-BiLSTM method is once again the winner with an accuracy of 84.53%, followed by Decision Trees with an accuracy of 82.93%. With an accuracy of 74.93%, Logistic Regression produced the lowest performance.

As illustrated in Table 6, we implemented Bangla-BERT for embedding and all previous algorithms for sentiment classification. As can be seen from the table, the CNN-BiLSTM approach wins with an accuracy of 94.15%, which exceeds all previous scores for other embedding methods and places this model above all previous models. SVM comes in second with an accuracy of 92.83%. Naïve Bayes performed the worst, with an accuracy of 87.81%.

Word2vec, Glove with LSTM classification performs almost identically in the challenge; however, Fasttext improves by 4% with an 88.35% score using the impact learning method. However, fine-tuned Bangla BERT with the LSTM classification model outperforms it. These experimental findings indicated that fine-tuning Bangla BERT with LSTM and CNN resulted in great improvement compared to the other embedding approach. As the improvement is much better, it reveals possibilities for subsequent advancements.

### 6.2. Comparison to Other Methods

• Hate Speech detection is one of the applications of sentiment analysis. To perform hate speech detection, Ref. [59] have used this dataset. The dataset contains five types of hate speech categories that include 35,000 statements in total. These are Political, Religious, Gender abusive, Geopolitical, and Personal. The total words present in the dataset are 672,109. They evaluated the hate speech detection dataset with BengFastText, Glove, and word2vec.
  In Table 7, we have incorporated the result of hate speech detection [59] and have performed a model averaging ensemble (MAE) on BengFastText, Glove, and word2vec. Their BengFastText shows the best F1 score of 0.891 in MAE, which is 1.3% better than the Multichannel Convolutional- LSTM (MC-LSTM) classifier. The Glove has an F1 score of 0.837 with MAE, which shows a 1.6% improvement over the MC-LSTM method. Word2vec showed the best result with a gradient boost classifier of 0.810, more influential than the MAE classifier result. Bangla-BERT significantly outperforms all of these, achieving a 0.941 F1 score. It exceeds BengFastText(MAE) by 0.05, giving a boost of 5%.
The Sentiment Analysis in the SAIL Dataset [60] contains tweets generated from the Shared Task on Sentiment Analysis in Indian Languages (SAIL) 2015. The dataset’s training, development, and test sets include 1000, 500, and 500 tweets, respectively.
The ABSA dataset [61] was created to facilitate aspect-based sentiment analysis tasks in Bangla. The dataset is divided into two sections: cricket and restaurant.
• The third dataset, the BengFastText dataset [59], was compiled from various sources, including newspapers, broadcast media, textbooks, websites, and social media. The original dataset contains 320,000 records that can be used for sentiment analysis. However, just a tiny portion of it is publicly accessible. For example, there are 8420 postings in the public version and the test set.
• On the other hand, the YouTube Comments Dataset [62] was created by collecting opinions from different YouTube videos. Three-class, five-class, and emotion labels were used to label the dataset. The 2796 comments were divided into train, development, and test sets.
• The final dataset for this comparison research is Social Media Posts (CogniSenti Dataset). It contains tweets and posts from Facebook and Twitter.

Table 8 denotes the classification score for each dataset. Among the traditional techniques, SVM beats RF, excluding the ABSA cricket dataset, where RF outperforms SVM by 2.6%. Deep learning models provide continuous improvements. Both Skip-Gram (Word2Vec) and CNN glove embedding perform comparably well. FastText performs better in three datasets than CNN does in others, while, ultimately, FastText outperforms CNN. Models based on transformers, such as BERT (Multilingual version), have gradually surpassed FastText. However, it indicates a reduction of 0.6% in the ABSA cricket dataset. Here, the suggested Bangla-BERT dataset outperforms all prior results, except the BengFastText dataset. Bangla-BERT outperforms all other techniques in terms of average F1 score, establishing this transformer-based model as the state-of-the-art approach compared to all other methods.

## 7. Conclusions and Future Work

This paper compares and contrasts various machine learning and deep learning algorithms for classifying texts according to their topics. We have demonstrated how transfer learning, the new revolution in natural language processing, can surpass all previous architectures. We have shown that transformer models such as BERT with proper fine-tuning can play a crucial role in sentiment analysis. Additionally, a CNN architecture was developed for this classification task. A very reliable pre-trained model was prepared for ease of use and made accessible as a python open-source package. Due to the fact that deep learning takes a rather large amount of data, we will continue to work on expanding the dataset. Additionally, we want to provide a Python API compatible with any web framework. We wish to use a particular word extraction algorithm to analyze the truth word extraction system for Bangla topic classification. We discovered that combining Bangla-BERT and LSTM leads to an astounding accuracy of 94.15%. However, LSTM gives the most significant overall result of all four-word embedding systems. We worked with an unbalanced dataset. A well-balanced dataset improves efficiency significantly. We want to use the sophisticated deep learning algorithm on a more enriched and balanced dataset in the future. Additionally, we offer an approach for assessing the performance of the proposed model in real-world applications.

## Figures and Tables

**Table 1:** Example of collected texts for sentiment analysis.

Text	Sentiment
জয় বাংলা কাপ! আর মার্চ মাস স্বাধীনতার মাস এমন একটি চমৎকার আইডিয়া যিনি নিয়ে এসেছেন তাকে স্যালুট (Joy Bangla Cup! And March is the month of independence. Salute to the one who came up with such a wonderful idea)	Positve
ক্লিনিকের মালিককে হোয়াইটওয়াশ করা দরকার (The owner of the clinic needs to be whitewashed)	Negative
কি বলব, দুই দিকেই সমস্যা আছে (What can I say, there are problems on both sides)	Negative
একটি সুন্দর সামাজিক পেজ পেইজে লাইক দিয়ে সাথেই থাকুন (A Beautiful social page. Stay by liking the page)	Positive

**Table 2:** Data pre-processing methods, step by step.

Processing	Text
Original	আমরা কাজ করবো কিভাবে! Document তৈরী করতে আমাদের সবাইকে কি করতে হবে ? 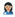 ৫-৬ জন আমরা,কঠিন হবে | ( How we work! What do we all have to do to create the document? 5-6 of us, it will be difficult )
Removing Punctuation	আমরা কাজ করবো কিভাবে Document তৈরী করতে আমাদের সবাইকে কি করতে হবে 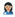 ৫ ৬ জন আমরা কঠিন হবে ( We will work on how to create a document We five six person will be difficult )
Removing Digits	আমরা কাজ করবো কিভাবে Document তৈরী করতে আমাদের সবাইকে কি করতে হবে 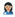 জন আমরা কঠিন হবে ( We will work out how to create a document what we all have to do John we will be difficult )
Removing Non-Bangla Character	আমরা কাজ করবো কিভাবে তৈরী করতে আমাদের সবাইকে কি করতে হবে 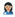 জন আমরা কঠিন হবে ( We will work hard to create what we all need to do )
Removing Emoticons	আমরা কাজ করবো কিভাবে তৈরী করতে আমাদের সবাইকে কি করতে হবে জন আমরা কঠিন হবে ( We will work hard to create what we all need to do )
Removing Stopwords	কাজ করবো কিভাবে তৈরী জন আমরা কঠিন হবে ( How will we work We will be difficult )

**Table 3:** Performance of Word2Vec.

Algorithms	Accuracy	Precision	Recall	F1 Score	ROC AUC	Kappa
SVM	0.8383	0.8791	0.7786	0.8287	0.8789	0.7111
Random Forest Tree	0.8184	0.8780	0.7239	0.8008	0.8984	0.6613
Logistic Regression	0.8025	0.8829	0.7026	0.7927	0.8831	0.6915
Naive Bayes	0.7816	0.8686	0.6418	0.7552	0.7776	0.4875
KNN	0.7946	0.8689	0.6001	0.7342	0.8444	0.7131
LDA	0.8138	0.8538	0.7739	0.8137	0.8438	0.6837
Decision Trees	0.8061	0.8526	0.6582	0.7559	0.8561	0.7259
LSTM	0.8281	0.8614	0.7352	0.7983	0.8284	0.7583
CNN	0.8188	0.8438	0.7706	0.8074	0.8999	0.7374
Impact Learning	0.8235	0.9114	0.7283	0.8197	0.8939	0.7697
ANN	0.7684	0.8426	0.7221	0.7829	0.8213	0.7129
CNN-BiLSTM	**0.8493**	0.9350	0.7248	0.8299	0.8131	0.6523

**Table 4:** Performance of fastText.

Algorithms	Accuracy	Precision	Recall	F1 Score	ROC AUC	Kappa
SVM	0.8483	0.8991	0.7586	0.8287	0.8989	0.7911
Random Forest Tree	0.8381	0.8280	0.8148	0.8201	0.8999	0.6813
Logistic Regression	0.8025	0.8829	0.7014	0.7927	0.8831	0.6915
Naive Bayes	0.7816	0.8616	0.6418	0.7552	0.7776	0.4875
KNN	0.7664	0.8480	0.6402	0.7442	0.8344	0.7101
LDA	0.8638	0.8838	0.7836	0.8337	0.9138	0.7172
Decision Trees	0.8461	0.8521	0.8005	0.8259	0.9061	0.7825
ANN	0.8381	0.8914	0.7096	0.7999	0.8884	0.7683
LSTM	0.8288	0.8431	0.7937	0.8174	0.8799	0.7474
CNN-BiLSTM	**0.8835**	0.9114	0.8098	0.8597	0.9319	0.7897
CNN	0.7684	0.8426	0.7262	0.7829	0.8213	0.7129
Impact Learning	0.8551	0.8738	0.7748	0.8249	0.8395	0.6523

**Table 5:** Performance of GloVe.

Algorithms	Accuracy	Precision	Recall	F1 Score	ROC AUC	Kappa
SVM	0.8252	0.8791	0.7492	0.8125	0.8789	0.7572
Random Forest Tree	0.8084	0.8282	0.7567	0.7908	0.8236	0.6613
Logistic Regression	0.7493	0.7923	0.6198	0.7035	0.7723	0.6915
Naive Bayes	0.7620	0.7826	0.6993	0.7402	0.8676	0.6237
KNN	0.7969	0.8592	0.6412	0.7492	0.8302	0.7294
LDA	0.8329	0.8523	0.7809	0.8147	0.8421	0.7837
Decision Trees	0.8293	0.8191	0.7639	0.7893	0.8419	0.7392
ANN	0.8181	0.8712	0.7494	0.8083	0.8329	0.7883
LSTM	0.8089	0.8330	0.7698	0.7974	0.8949	0.7371
Impact Learning	0.8262	0.8923	0.7786	0.8337	0.8925	0.7797
CNN	0.7884	0.8220	0.6253	0.7229	0.8113	0.7629
CNN-BiLSTM	**0.8453**	0.9649	0.7048	0.8349	0.8135	0.6323

**Table 6:** Performance of Bangla-BERT.

Algorithms	Accuracy	Precision	Recall	F1 Score	ROC AUC	Kappa
SVM	0.9283	0.9391	0.9282	0.9287	0.9389	0.92111
Random Forest Tree	0.9184	0.9380	0.8948	0.9108	0.9284	0.93613
Logistic Regression	0.9025	0.8982	0.8725	0.8827	0.8831	0.86915
Naive Bayes	0.8781	0.8821	0.8641	0.8755	0.8777	0.7487
KNN	0.8794	0.8868	0.8235	0.8534	0.8844	0.7931
LDA	0.9013	0.8853	0.8544	0.8713	0.8683	0.7683
Decision Trees	0.9006	0.8952	0.8389	0.8655	0.8956	0.8725
ANN	0.9120	0.9067	0.8954	0.8998	0.8958	0.9063
CNN	0.9108	0.9243	0.9001	0.9077	0.9349	0.9527
Impact Learning	0.8833	0.8931	0.8651	0.8770	0.8994	0.8061
LSTM	0.9148	0.9159	0.8692	0.8918	0.8881	0.8762
CNN-BiLSTM	**0.9415**	0.9423	0.9294	**0.9304**	**0.9473**	**0.9222**

**Table 7:** Performance of hate speech detection.

Method	Classifier	Precision	Recall	F1
BengFastText	MC-LSTM	0.881	0.883	0.882
BengFastText	MAE	0.894	0.896	0.891
GloVe	MC-LSTM	0.827	0.822	0.824
GloVe	MAE	0.831	0.834	0.837
Word2vec	GBT	0.806	0.815	0.810
Word2vec	MAE	0.79	0.800	0.790
Bangla-BERT	CNN-BiLSTM	0.946	0.949	**0.941**

**Table 8:** Classification results using RF, SVM, CNN, FastText, BERT, and Bangla-BERT.

Dataset	RF	SVM	CNN-W2V	CNN-GLOVE	Fast-Text	BERT	Bangla-BERT
ABSA cricket	0.662	0.636	0.679	0.696	0.688	0.682	0.698
ABSA Restaurant	0.407	0.498	0.391	0.491	0.519	0.581	0.701
SAIL	0.546	0.552	0.557	0.595	0.532	0.566	0.604
BengFastText	0.612	0.613	0.663	0.657	0.661	0.674	0.672
Youtube comments	0.586	0.605	0.669	0.663	0.658	0.729	0.741
CogniSenti	0.545	0.584	0.604	0.587	0.614	0.686	0.681
Avg.	0.560	0.581	0.594	0.615	0.612	0.653	**0.683**

## Data Availability

Not applicable.

## References

[1] Kowsher M., Afrin F., Sanjid Z.I. Machine Learning and Deep Learning-Based Computing Pipelines for Bangla Sentiment Analysis. Proceedings of the International Joint Conference on Advances in Computational Intelligence.

[2] Kowsher M., Tahabilder A., Sanjid M.Z.I., Prottasha N.J., Sarker M.M.H. Knowledge-base optimization to reduce the response time of bangla chatbot. Proceedings of the 2020 Joint 9th International Conference on Informatics, Electronics & Vision (ICIEV) and 2020 4th International Conference on Imaging, Vision & Pattern Recognition (icIVPR).

[3] Rogers A., Kovaleva O., Rumshisky A. (2020). A primer in bertology: What we know about how bert works. Trans. Assoc. Comput. Linguist..

[4] Devlin J., Chang M.W., Lee K., Toutanova K. (2018). Bert: Pre-training of deep bidirectional transformers for language understanding. arXiv.

[5] Libovickỳ J., Rosa R., Fraser A. (2019). How language-neutral is multilingual BERT?. arXiv.

[6] Kowsher M., Uddin M.J., Tahabilder A., Amin M.R., Shahriar M.F., Sobuj M.S.I. BanglaLM: Data Mining based Bangla Corpus for Language Model Research. Proceedings of the 2021 Third International Conference on Inventive Research in Computing Applications (ICIRCA).

[7] Dashtipour K., Gogate M., Li J., Jiang F., Kong B., Hussain A. (2020). A hybrid Persian sentiment analysis framework: Integrating dependency grammar based rules and deep neural networks. Neurocomputing.

[8] Kennedy A., Inkpen D. (2006). Sentiment classification of movie reviews using contextual valence shifters. Comput. Intell..

[9] Cui H., Mittal V., Datar M. (2006). Comparative Experiments on Sentiment Classification for Online Product Reviews.

[10] Kouloumpis E., Wilson T., Moore J. Twitter sentiment analysis: The good the bad and the omg! In Proceedings of the 5th International AAAI Conference on Weblogs and Social Media.

[11] Krishna A., Akhilesh V., Aich A., Hegde C. (2019). Sentiment analysis of restaurant reviews using machine learning techniques. Emerging Research in Electronics, Computer Science and Technology.

[12] Singla Z., Randhawa S., Jain S. Sentiment analysis of customer product reviews using machine learning. Proceedings of the 2017 International Conference on Intelligent Computing and Control (I2C2).

[13] Souza M., Vieira R. Sentiment analysis on twitter data for portuguese language. Proceedings of the International Conference on Computational Processing of the Portuguese Language.

[14] Ombabi A.H., Ouarda W., Alimi A.M. (2020). Deep learning CNN–LSTM framework for Arabic sentiment analysis using textual information shared in social networks. Soc. Netw. Anal. Min..

[15] Mathews D.M., Abraham S. Social data sentiment analysis of a multilingual dataset: A case study with malayalam and english. Proceedings of the International Conference on Advanced Informatics for Computing Research.

[16] Chowdhury R.R., Hossain M.S., Hossain S., Andersson K. Analyzing sentiment of movie reviews in bangla by applying machine learning techniques. Proceedings of the 2019 International Conference on Bangla Speech and Language Processing (ICBSLP).

[17] Paul A.K., Shill P.C. Sentiment mining from bangla data using mutual information. Proceedings of the 2nd International Conference on Electrical, Computer & Telecommunication Engineering (ICECTE).

[18] Hossain E., Sharif O., Moshiul Hoque M. (2021). Sentiment polarity detection on bengali book reviews using multinomial naive bayes. Progress in Advanced Computing and Intelligent Engineering.

[19] Mahtab S.A., Islam N., Rahaman M.M. Sentiment analysis on bangladesh cricket with support vector machine. Proceedings of the 2018 International Conference on Bangla Speech and Language Processing (ICBSLP).

[20] Chowdhury S., Chowdhury W. Performing sentiment analysis in Bangla microblog posts. Proceedings of the 2014 International Conference on Informatics, Electronics & Vision (ICIEV).

[21] Peters M.E., Neumann M., Iyyer M., Gardner M., Clark C., Lee K., Zettlemoyer L. (2018). Deep contextualized word representations. arXiv.

[22] Brown T.B., Mann B., Ryder N., Subbiah M., Kaplan J., Dhariwal P., Neelakantan A., Shyam P., Sastry G., Askell A. (2020). Language models are few-shot learners. arXiv.

[23] Qiu X., Sun T., Xu Y., Shao Y., Dai N., Huang X. (2020). Pre-trained models for natural language processing: A survey. arXiv.

[24] Dai X., Karimi S., Hachey B., Paris C. (2020). Cost-effective selection of pretraining data: A case study of pretraining BERT on social media. arXiv.

[25] Antoun W., Baly F., Hajj H. (2020). Arabert: Transformer-based model for arabic language understanding. arXiv.

[26] Farahani M., Gharachorloo M., Farahani M., Manthouri M. (2021). Parsbert: Transformer-based model for persian language understanding. Neural Process. Lett..

[27] de Vries W., van Cranenburgh A., Bisazza A., Caselli T., van Noord G., Nissim M. (2019). Bertje: A dutch bert model. arXiv.

[28] Masala M., Ruseti S., Dascalu M. Robert–a romanian bert model. Proceedings of the 28th International Conference on Computational Linguistics.

[29] Kuratov Y., Arkhipov M. (2019). Adaptation of deep bidirectional multilingual transformers for russian language. arXiv.

[30] Cui Y., Che W., Liu T., Qin B., Yang Z., Wang S., Hu G. (2019). Pre-training with whole word masking for chinese bert. arXiv.

[31] Kowsher M., Sobuj M.S.I., Shahriar M.F., Prottasha N.J., Arefin M.S., Dhar P.K., Koshiba T. (2022). An Enhanced Neural Word Embedding Model for Transfer Learning. Appl. Sci..

[32] Kowsher M., Uddin M.J., Tahabilder A., Prottasha N.J., Ahmed M., Alam K.R., Sultana T. (2021). BnVec: Towards the Development of Word Embedding for Bangla Language Processing. Int. J. Eng. Technol..

[33] Hohman F., Wongsuphasawat K., Kery M.B., Patel K. Understanding and visualizing data iteration in machine learning. Proceedings of the 2020 CHI Conference on Human Factors in Computing Systems.

[34] Mridha M.F., Banik M., Ali M.N.Y., Huda M.N., Rahman C.M., Das J.K. Formation of Bangla Word Dictionary Compatible with UNL Structure. Proceedings of the 4th International Conference on Software, Knowledge, Information Management and Applications (SKIMA).

[35] Misra J. (2020). AutoNLP: NLP feature recommendations for text analytics applications. arXiv.

[36] Gupta S., Kanchinadam T., Conathan D., Fung G. (2020). Task-optimized word embeddings for text classification representations. Front. Appl. Math. Stat..

[37] Grohe M. word2vec, node2vec, graph2vec, x2vec: Towards a theory of vector embeddings of structured data. Proceedings of the 39th ACM SIGMOD-SIGACT-SIGAI Symposium on Principles of Database Systems.

[38] Shobana J., Murali M. (2021). Improving feature engineering by fine tuning the parameters of Skip gram model. Mater. Today Proc..

[39] Choudhari P., Veenadhari S. (2020). Sentiment Classification of Online Mobile Reviews Using Combination of Word2vec and Bag-of-Centroids. Machine Learning and Information Processing.

[40] Sakketou F., Ampazis N. (2020). A constrained optimization algorithm for learning GloVe embeddings with semantic lexicons. Knowl. Based Syst..

[41] Selva Birunda S., Kanniga Devi R. (2021). A Review on Word Embedding Techniques for Text Classification. Innovative Data Communication Technologies and Application.

[42] Mikolov T., Chen K., Corrado G., Dean J. (2013). Efficient estimation of word representations in vector space. arXiv.

[43] Vaswani A., Shazeer N., Parmar N., Uszkoreit J., Jones L., Gomez A.N., Kaiser Ł., Polosukhin I. Attention is all you need. Proceedings of the Advances in Neural Information Processing Systems 30 (NIPS 2017).

[44] Rush A.M. The annotated transformer. Proceedings of the Workshop for NLP Open Source Software (NLP-OSS).

[45] Schmidhuber J. (2015). Deep Learning in Neural Networks: An Overview. Neural Netw..

[46] LeCun Y., Boser B., Denker J.S., Henderson D., Howard R.E., Hubbard W., Jackel L.D. (1989). Backpropagation Applied to Handwritten Zip Code Recognition. Neural Comput..

[47] Acharya U.R., Oh S.L., Hagiwara Y., Tan J.H., Adam M., Gertych A., Tan R.S. (2017). A deep convolutional neural network model to classify heartbeats. Comput. Biol. Med..

[48] Dmitrievich I.A. (2015). Deep Learning in Information Analysis of Electrocardiogram Signals for Disease Diagnostics. Bachelor’s Thesis.

[49] Zubair M., Kim J., Yoon C. An Automated ECG Beat Classification System Using Convolutional Neural Networks. Proceedings of the 2016 6th International Conference on IT Convergence and Security (ICITCS).

[50] Pourbabaee B., Roshtkhari M.J., Khorasani K. (2018). Deep Convolutional Neural Networks and Learning ECG Features for Screening Paroxysmal Atrial Fibrillation Patients. IEEE Trans. Syst. Man Cybern. Syst..

[51] Gers F.A., Schmidhuber J., Cummins F. Learning to forget: Continual prediction with LSTM. Proceedings of the 1999 9th International Conference on Artificial Neural Networks ICANN 99. (Conf. Publ. No. 470).

[52] Kowsher M., Tahabilder A., Sanjid M.Z.I., Prottasha N.J., Uddin M.S., Hossain M.A., Jilani M.A.K. (2021). LSTM-ANN & BiLSTM-ANN: Hybrid deep learning models for enhanced classification accuracy. Procedia Comput. Sci..

[53] Yildirim Ö. (2018). A novel wavelet sequence based on deep bidirectional LSTM network model for ECG signal classification. Comput. Biol. Med..

[54] Graves A., Schmidhuber J. (2005). Framewise phoneme classification with bidirectional LSTM and other neural network architectures. Neural Netw..

[55] De Baets L., Ruyssinck J., Peiffer T., Decruyenaere J., De Turck F., Ongenae F., Dhaene T. (2016). Positive blood culture detection in time series data using a BiLSTM network. arXiv.

[56] Khan Mamun M.M.R., Alouani A.T. FA-1D-CNN Implementation to Improve Diagnosis of Heart Disease Risk Level. Proceedings of the 6th World Congress on Electrical Engineering and Computer Systems and Sciences (EECSS’20), Virtual Conference.

[57] Kulkarni A., Mandhane M., Likhitkar M., Kshirsagar G., Joshi R. (2021). L3cubemahasent: A marathi tweet-based sentiment analysis dataset. arXiv.

[58] Nguyen Q.T., Nguyen T.L., Luong N.H., Ngo Q.H. Fine-tuning bert for sentiment analysis of vietnamese reviews. Proceedings of the 7th NAFOSTED Conference on Information and Computer Science (NICS).

[59] Karim M.R., Chakravarthi B.R., McCrae J.P., Cochez M. Classification benchmarks for under-resourced bengali language based on multichannel convolutional-lstm network. Proceedings of the IEEE 7th International Conference on Data Science and Advanced Analytics (DSAA).

[60] Patra B.G., Das D., Das A., Prasath R. Shared task on sentiment analysis in indian languages (sail) tweets-an overview. Proceedings of the International Conference on Mining Intelligence and Knowledge Exploration.

[61] Rahman M., Kumar Dey E. (2018). Datasets for aspect-based sentiment analysis in bangla and its baseline evaluation. Data.

[62] Tripto N.I., Ali M.E. Detecting multilabel sentiment and emotions from bangla youtube comments. Proceedings of the 2018 International Conference on Bangla Speech and Language Processing (ICBSLP).

